# Portal hypertension in common variable immunodeficiency disorders – a single center analysis on clinical and immunological parameter in 196 patients

**DOI:** 10.3389/fimmu.2023.1268207

**Published:** 2023-12-20

**Authors:** Leif G. Hanitsch, Sophie Steiner, Michael Schumann, Kirsten Wittke, Claudia Kedor, Carmen Scheibenbogen, Andreas Fischer

**Affiliations:** ^1^ Institute of Medical Immunology, Charité - Universitätsmedizin Berlin, Corporate Member of Freie Universität Berlin and Humboldt Universität zu Berlin, Berlin Institute of Health, Berlin, Germany; ^2^ Berlin Institute of Health at Charité - Universitätsmedizin Berlin, BIH Center for Regenerative Therapies (BCRT), Berlin, Germany; ^3^ Department of Gastroenterology, Infectiology and Rheumatology, Charité-Universitätsmedizin Berlin, Corporate Member of Freie Universität Berlin and Humboldt-Universität zu Berlin and Berlin Institute of Health, Berlin, Germany; ^4^ Department of Internal Medicine and Gastroenterology, Caritas-Klinik Maria Heimsuchung Berlin-Pankow, Berlin, Germany; ^5^ Department of Hepatology and Gastroenterology, Charité - Universitätsmedizin Berlin, Corporate Member of Freie Universität Berlin and Humboldt Universität zu Berlin, Berlin Institute of Health, Berlin, Germany

**Keywords:** common variable immunodeficiency disorder (CVID), primary immunodeficiency (PID), inborn errors of immunity (IEI), liver, portal hypertension (PHT), nodular regenerative hyperplasia-like changes (NRH-LC)

## Abstract

**Background:**

Liver manifestations and in particular portal hypertension (PH) contribute significantly to morbidity and mortality of patients with common variable immunodeficiency disorders (CVID). Screening strategies and early detection are limited due to the lack of specific diagnostic tools.

**Methods:**

We evaluated clinical, immunological, histological, and imaging parameters in CVID patients with clinical manifestation of portal hypertension (CVID+PH).

**Results:**

Portal hypertension was present in 5.6% of CVID patients and was associated with high clinical burden and increased mortality (18%). Longitudinal data on clinical and immunological parameters in patients before and during clinically manifest portal hypertension revealed a growing splenomegaly and increasing gamma-glutamyl transferase (GGT) and soluble interleukin 2 receptor (SIL-2R) levels with decreasing platelets over time. While ultrasound of the liver failed to detect signs of portal hypertension in most affected patients, transient elastography was elevated in all patients. All CVID+PH patients had reduced naïve CD45RA+CD4+ T-cells (mean of 6,2%). The frequency of severe B-lymphocytopenia (Euroclass B-) was higher in CVID+PH patients. The main histological findings included lymphocytic infiltration, nodular regenerative hyperplasia-like changes (NRH-LC), and porto(-septal) fibrosis.

**Conclusion:**

CVID patients with lower naïve CD45RA+CD4+ T-cells or severely reduced B-cells might be at higher risk for portal hypertension. The combination of biochemical (increasing sIL-2R, GGT, and decreasing platelets) and imaging parameters (increasing splenomegaly) should raise suspicion of the beginning of portal hypertension.

## Introduction

Common variable immunodeficiency disorders (CVID) comprise the largest clinically relevant group of primary immunodeficiency disorders (PID) with an estimated prevalence of 1:25.000 (1). According to the European Society for Immunodeficiencies (ESID) criteria, patients present with relevant IgG and IgA +/- IgM deficiency together with reduced class-switched memory B cells and/or impaired specific antibody response to vaccination. There is substantial clinical heterogeneity in CVID patients as a result of infectious as well as non-infectious complications. In particular, non-infectious manifestations contribute significantly to disease morbidity and mortality and may present as immune cytopenia, lymphoproliferation, granulomatous disease, interstitial lung disease, enteropathy, autoimmunity, malignancy, or other signs of immune dysregulation (2).

The clinical burden and mortality of CVID-related liver diseases is high (3–6), however, data on prevalence vary substantially between 9% and 79% and depend greatly on the defining inclusion criteria (7). Considering elevated liver enzymes, approximately 50% of CVID patients were found to present with any sort of hepatic manifestation (8, 9), however, histologically proven liver involvement was reported to occur in only 9% of patients (10). 

In recent publications, approximately 4-6% of adult CVID patients were found to suffer from clinically relevant portal hypertension (4, 11), presenting with non-malignant ascites, esophageal varices, or other signs of increased portal pressure and expressing an elevated mortality rate as high as 41% (4).

Most CVID patients with portal hypertension were reported to suffer from additional non-hepatic organ manifestations, such as autoimmune cytopenia, enteropathy, granulomatous disease, interstitial lung disease, and lymphoproliferation (4), but it remains unclear if these overlapping non-infectious manifestations follow common pathomechanisms. While other non-infectious complications may occur early or even before CVID diagnosis, portal hypertension is regarded as a late organ manifestation (4). Although the majority of patients with portal hypertension show signs of nodular regenerative hyperplasia (NRH) in histopathology (7, 12, 13), there is significant heterogeneity and potential risk factors remain incompletely understood. Due to the parallel appearance of nodular regenerative hyperplasia together with other histopathological features, Crotty et al. coined the term NHR-like changes (NRH-LC), which will be used in this article (13).

On a cellular level, hepatic disease in CVID is associated with (CD8+) T cell infiltration, while only a few B cells are detectable in the liver tissue of affected patients (12, 13).

In order to avoid delayed diagnosis of portal hypertension, screening strategies for early detection are important (14). Splenomegaly and elevated liver stiffness were proposed to be associated with portal hypertension (4), however, changes in these parameters can result for multiple reasons and invasive measurement of the hepatic venous pressure gradient (HVPG) may be required to establish the diagnosis.

In the present study, we retrospectively analyzed the occurrence of portal hypertension in a single-center cohort of 196 adult CVID patients. We identified 11 patients (5.6%) who presented with non-malignant ascites, esophageal varices, or gastropathy as clinical manifestations of significant portal hypertension. We compare clinical and immunological data between affected (CVID+PH) and non-affected patients and report on liver histology as well as on treatment of portal hypertension. In addition, we provide longitudinal laboratory and imaging data of eight affected patients before developing and while suffering from portal hypertension.

## Methods

### Patients

All patients fulfilled the ESID criteria for CVID and were treated and followed regularly at the Immunodeficiency Outpatient Clinic at Charité Universitaetsmedizin Berlin, Germany. Patients with CVID and PH were identified in the data information system in a retrospective manner. Clinically relevant PH was defined as the presence of esophageal varices and signs of portal hypertensive gastropathy or ascites. In total, 12 of 196 CVID patients fulfilled the definition of clinically relevant PH. After the exclusion of one patient due to plausible underlying conditions other than CVID-related liver manifestations (the patient presented with a history of hepatitis C and long-term drug treatment with known hepatotoxic potential), a total of 11 patients (5.6%) were included in the present study. All patients received regular immunoglobulin replacement treatment.

The study was approved by the local ethics committee, and written informed consent was obtained from all individual participants included in the study.

### Non-invasive imaging

Data from ultrasound in B-mode and with Doppler as well as data from vibration-controlled transient elastography (FibroScan^®^) were included in this study. FibroScan^®^ uses transducer-induced vibrations that result in the movement of shear waves. Calculations of the velocity of the shear waves in liver parenchyma allow one to estimate the degree of liver stiffness (15). The measurements were conducted by an experienced ultrasonographer who completed 10 serial measurements with the median score being reported.

### Invasive measurements

Where available, data from interventional radiology measuring hepatic venous pressure gradient (HVPG) were included. All measurements were conducted at the local Department of Radiology as a routine examination.

### Histology

Liver samples were obtained by transjugular puncture (five samples from five patients) or by sonography-guided percutaneous puncture (five samples from three patients). Two patients received additional T-cell receptor (TCR) clonality assays using a BIOMED II Primer panel and following EuroClonality/BIOMED-2 guidelines for analysis and interpretation (16).

### Immunological and other liver-related laboratory parameter

Basic immunological parameters included IgG, IgA, IgM, soluble interleukin 2 receptor (sIL-2R = sCD25), CD3^+^, CD4^+^, CD8^+^, CD45RA, CD45RO, and CD19^+^ cells as well as NK cells. B cells were further analyzed according to the EUROClass trial. B cell subsets could not be determined in 12/196 CVID patients due to complete B lymphopenia. Other liver-related laboratory parameters included aspartate aminotransferase (AST), alanine transaminase (ALT), bilirubin, gamma-glutamyl transferase (GGT), international normalized ratio (INR), activated partial thromboplastin time (aPTT), and levels of thrombocytes. In addition, CVID+PH patients were tested for the presence of autoantibodies anti-nuclear antibodies (ANA), antimitochondrial antibodies (AMA), and Anti-mitochondrial M2 antibody (AMA-M2), and PCR testing for HBV, HCV, and HEV (hepatitis B, C, and E) was conducted.

### Data analysis

GraphPad Prism version 9.3.1 was used for statistical analyses. The statistical tests used are indicated in the figure legends. A p-value of < 0.05 was considered statistically significant.

## Results

We identified 11 patients with symptomatic PH. Symptom onset occurred with a median delay of 7 years (IQR: 4.5 years; mean delay 10 years) and ranging from 3 years to 36 years after establishing CVID diagnosis. In our cohort, 9/11 patients were female (see **Table 1**). The mean observation period for all patients was 12 years (IQR: 2 years).

**Table 1:** Data on treatment and outcome of portal hypertension in CVID+PH (n: 11).

	Onset of portal hypertension after CVID diagnosis	Conservative treatment	Invasive treatment	Other treatments for liver manifestations	Outcome
**CVID+PH (#1)**	7 years	Carvedilol	single ascites paracentesis, recurrent banding for esophageal varices	splenectomy	alive
**CVID+PH (#2)**	10 years	Carvedilol, diuretics	No	no	alive
**CVID+PH (#3)**	10 years	Carvedilol, diuretics	recurrent banding for esophageal varices	no	alive
**CVID+PH (#4)**	7 years	Carvedilol	No	Rituximab (massive splenomegaly)	alive
**CVID+PH (#5)**	22 years	Carvedilol, diuretics	recurrent ascites paracentesis, recurrent banding for esophageal varices	treatment with TNF alpha inhibitor (adalimumab), splenectomy	dead
**CVID+PH (#6)**	6 years	Carvedilol, diuretics	TIPS, recurrent ascites paracentesis	antibiotic prophylaxis with norfloxacin	alive (acute renal failure)
**CVID+PH (#7)**	6 years	Carvedilol, diuretics	TIPS, recurrent ascites paracentesis	MMF, steroids, antibiotic prophylaxis with rifaximin, lactulose, in preparation for dialysis due to hepato-renal syndrome	alive (hepatic encephalopathy, septic shock after TIPS)
**CVID+PH (#8)**	3 years	no	No	no	alive
**CVID+PH (#9)**	5 years	no	No	splenectomy	alive
**CVID+PH (#10)**	3 years	Carvedilol, diuretics	recurrent ascites paracentesis, recurrent banding for esophageal varices	Abatacept 125mg 2x/wk	dead (spontaneous bacterial peritonitis)
**CVID+PH (#11)**	36 years	Carvediolol, diuretics	TIPS, recurrent ascites paracentesis, recurrent banding for esophageal varices	Rifaximin, lactulose	alive (spontaneous bacterial peritonitis, hepatic encephalopathy)

CVID, Common Variable Immunodeficiency Disorder; MMF, mycophenolat mofetil; PH, portal hypertension; TIPS, transjugular intrahepatic portosystemic shunt; TNF, tumor necrisis factor; wk, week.

### Clinic

Of the eleven patients who presented with clinical signs of PH, five patients suffered from ascites, eight patients showed esophageal varices or portal hypertensive gastropathy in endoscopy (n:3), and seven patients were CVID affected from both (see **Supplementary Table S1**).

There was a significant association of CVID+PH patients with other non-infectious manifestations, in particular with granulomatous–lymphocytic interstitial lung disease (GLILD) (p-value < 0.0001) or extra-pulmonary granulomatous lesions (p-value < 0,009) as well as with splenomegaly (p-value < 0.0001) or lymphadenopathy (p-value < 0.0001). A clear classification of low platelets in patients with suspected hypersplenism is difficult. After careful reconsideration, we only rated two patients to be affected with immune thrombocytopenic purpura (ITP). Four additional patients were tested repeatedly with low thrombocytes between 50-100/nl but never required specific treatment for ITP. In the absence of anti-thrombocyte antibodies and assuming a pathophysiological process related to hypersplenism, we refrained from using the term ITP in these patients (**Table 2**).

**Table 2:** Demographic data and clinical manifestations in CVID patients with portal hypertension (CVID+PH) and CVID patients without portal hypertension (CVID).

	CVID+PH (n: 11)	CVID (n: 185)	p-value
Sex (female/male)	2/9	103/82	0.0887
Mean age (in years)	51	47	0.3077
AIHA	3/11	19/185	0.0826
ITP	2/11	31/185	0.9023
Thrombocytopenia due to hypersplenism	4/11	4/185	<0.00001
Enteropathy	5/11	40/185	0.0679
GLILD	9/11	30/185	<0.00001
Bronchiectasis	4/11	36/185	0.1765
Extrapulmonary granuloma	5/11	28/185	0.009
Splenomegaly	11/11	70/185	<0.00001
Lymphadenopathy	9/11	41/185	<0.00001

ITP refers only to patients with a history of severe thrombocytopenia requiring treatment. Thrombocytopenia due to hypersplenism includes all patients with splenomegaly and who repeatedly tested with reduced thrombocytes between 50-100/nl. The p-value was calculated using chi square test and two-tailed Mann-Whitney U Test. AIHA, autoimmune hemolytic anemia; CVID, Common Variable Immunodeficiency Disorder; GLILD, granulomatous–lymphocytic interstitial lung disease; ITP, immune thrombocytopenia; PH, portal hypertension.

### Genetics

Whole exome sequencing (WES) was conducted by next-generation sequencing (NGS) in all 11 patients suffering from portal hypertension (CVID+PH), revealing a pathogenic mutation for NFKB1 in patient #3 and CTLA4 haploinsufficiency in patient #10. No disease-causing mutation was detected in the remaining CVID+PH patients (see **Supplementary Table S1**).

### Management and outcome

Conservative management involved treatment with non-cardioselective beta-blocker carvedilol in 9/11 patients (82%) and diuretics in 7/11 patients (64%). Invasive procedures included paracentesis of ascites in 5/11 patients (45%), banding of esophageal varices in 5/11 patients (45%), and implantation of transjugular intrahepatic portosystemic shunt (TIPS) in 3/11 patients (27%). One patient with *CTLA4* haploinsufficiency (patient #10) had received abatacept with a starting dose of 125mg weekly and increased dosing of 2x 125mg weekly. Patient #5 was treated with a TNF alpha inhibitor due to the presence of hepatic granulomatous lesions. Treatment was started with infliximab and switched to adalimumab after the detection of anti-drug autoantibodies against infliximab. Patient #4 received two doses of 1000mg rituximab for treatment of massive splenomegaly. In short-term follow-up, the splenic pole-to-pole distance was reduced by 3-4cm.

Other significant aspects of management and complications included: septic shock shortly after TIPS placement (n:1), acute renal failure under intensified diuretic treatment (n:1), hepato-renal syndrome (n:1), spontaneous bacterial peritonitis (n:3), and hepatic encephalopathy (n:2). Three patients had received splenectomy (one patient before and two patients already suffering from PH). Two patients died during the observation period (18%). Patient #10 died of a septic shock after spontaneous bacterial peritonitis, and patient #5 died due to hemorrhagic shock after a failed intervention to place a lysis catheter for severe portal vein thrombosis and acute liver failure (see **Table 1**). Mortality in the CVID+PH patients was higher than in the CVID patients without PH (18% vs. 6%).

### Ultrasound (B-Mode and Doppler)

Ultrasound imaging was available for all 11 patients. Longitudinal ultrasound data was available for eight CVID+PH patients (data from 2-5 time points). In B-Mode, sonographic signs of chronic parenchymal liver damage were visible in 2/11 patients, 3/11 patients showed some changes in the homogeneity of hepatic parenchyma, and six patients had no detectable parenchymal liver changes in sonography at all.

Using Doppler, only the two patients with detectable chronic parenchymal liver damage showed reduced flow velocity in the portal trunk (peak systolic velocity <16 cm/sec), and the remaining 9 patients had normal flow velocities. Splenomegaly was present in all patients (two patients had undergone splenectomy) with a mean pole-to-pole distance of 22 cm (ranging from 16 to 28 cm in length) (see **Table 3**).

**Table 3:** Data on sonography, transient elastography (FibroScan), and invasive portal pressure measurements (HVPG).

	Ultrasound (B-mode)	Ultrasound (Doppler)	Transient elastography	Hepatic venous pressure gradient (HVPG)
**CVID+PH (#1)**	Mild hepatomegaly. No parenchymal abnormalities. Splenectomy in 2007 due to splenomegaly.	normal velocity in portal trunk	21 kPa	15 mmHg
**CVID+PH (#2)**	Mild hepatomegaly. Prominent splenic vein. No parenchymal abnormalities. Increasing splenomegaly (max. 21 cm pole-to-pole).	normal velocity in portal trunk	12.1 kPa	18 mmHg
**CVID+PH (#3)**	Mild hepatomegaly. Hypoechoic lesions. Otherwise no parenchymal abnormalities. Increasing splenomegaly (max. 19 cm pole-to-pole).	normal velocity in portal trunk	14.4 kPa	10 mmHg
**CVID+PH (#4)**	No parenchymal abnormalities. Increasing splenomegaly (max. 26 cm pole-to-pole).	normal velocity in portal trunk	20 kPa	29 mmHg
**CVID+PH (#5)**	Chronic parenchymal liver damage. Prominent splenic vein. Increasing splenomegaly (max. 28 cm pole-to-pole).	reduced velocity in portal trunk	n.d.	n.d.
**CVID+PH (#6)**	No parenchymal abnormalities. Increasing splenomegaly (max. 24 cm pole-to-pole).	normal velocity in portal trunk	12.4 kPa	23 mmHg
**CVID+PH (#7)**	Coarsened liver parenchyma. Increasing splenomegaly (max. 20 cm pole-to-pole).	normal velocity in portal trunk	25 kPa	20 mmHg
**CVID+PH (#8)**	No parenchymal abnormalities. Increasing splenomegaly (max. 16 cm pole-to-pole).	normal velocity in portal trunk	n.d.	n.d.
**CVID+PH (#9)**	Mild hepatomegaly. No parenchymal abnormalities. Splenectomy in 2021.	normal velocity in portal trunk	8.7 kPa	n.d.
**CVID+PH (#10)**	Inhomogeneous parenchyma. Prominent splenic vein. Distended portal vein (1,8 cm). Splenomegaly (26x9 cm).	normal velocity in portal trunk	9.4 kPa	n.d.
**CVID+PH (#11)**	Chronic parenchymal liver damage. Splenomegaly.	reduced velocity in portal trunk	31 kPa	28 mmHg

CVID, Common Variable Immunodeficiency Disorder; kPA, kilopascal; mmHg, millimetres of mercury; n.d., not defined; PH, portal hypertension.

### Transient elastography

Transient elastography (FibroScan^®^) is a non-invasive method proposed for the assessment of hepatic fibrosis in patients with chronic liver disease by measuring liver stiffness. Data were available for 9/11 patients with non-cirrhotic PH. Using a cut-off < 6,4 kPa as normal, all patients presented with elevated levels, ranging from mildly elevated (8.7 kPa ≙ fibrosis score F2) to very high levels (31 kPa ≙ liver fibrosis F4) (see **Table 3**).

### Invasive portal pressure measurement

HVPG was measured in seven patients (see **Table 3**). Despite normal velocity in Doppler examination, an elevated HVPG of at least 10 mmHg, which is defined as the cut-off for clinically significant PH, was detected in all patients. Mean HVPG was 20.4 mmHg (median 20 mmHg).

### Histopathology

Histopathology was available for 9/11 patients. The majority expressed histopathological changes of nodular regenerative hyperplasia-like changes (present in 4/9 patients) and lymphocytic infiltration (present in 7/9 patients). Histopathological findings of granulomatous lesions and portal/porto-septal fibrosis were present in two patients each (see **Table 4**).

**Table 4:** Data on histopathology in CVID+PH patients.

	Histopathology of liver
**CVID+PH (#1)**	No epiteloid granuloma, Kupffer cell hyperplasia, discrete lobular sinusoidal lymphocytic infiltration, 15% steatosis
**CVID+PH (#2)**	Mild portal and to a lesser degree acinar lymphocytic infiltration. Aspects of NRH. Portal and slight portoseptal fibrosis.
**CVID+PH (#3)**	NRH. Mild portal inflammation with biliary degeneration (Desmet Grade 1). Portoseptal Fibrosis (Desmet Grade 2). No steatosis.
**CVID+PH (#4)**	NRH and minimal, unspecific hepatitis. No steatosis.
**CVID+PH (#5)**	2014: discontinious portal hepatitis, discrete lymphocytic infiltration and infiltrative portal fibrosis. Microgranuloma and non-necrotizing granuloma. 2015: moderate to severe portal and lobular granulomatous inflammation and necrosis of hepatocytes. 2018: portal hepatitis, lymphoid infiltration, no granuloma. Activated hepatocytes. Irregularly distributed steatosis (<10%). Spleen 2019: Infiltrates of a nodular lymphocyte-predominant Non-Hodgkin-Lymphoma
**CVID+PH (#6)**	No significant portal inflammation with focal portoseptal fibrosis and mild acinar reaction. No steatosis. No cholangitis. No vasculitis. No granuloma. No siderosis. No lymphofollicular aggregates. Compatible with NRH.
**CVID+PH (#7)**	NRH and lobular T lymphocytic infiltration (oligoclonal in TCR typing)
**CVID+PH (#8)**	No histology available
**CVID+PH (#9)**	lymphocyctic infiltration, minimal interface-hepatitis, few epitheloid cell granuloma, slight periportal and perisinusoidal fibrosis
**CVID+PH (#10)**	No histology available
**CVID+PH (#11)**	Extensive CD8+ T lymphocytic infiltration (oligoclonal in TCR typing)

CVID, Common Variable Immunodeficiency Disorder; NRH, nodular regenerative hyperplasia; TCR, T cell receptor.

Over the period of 5 years, patient #5 had received three liver biopsies. The first biopsy took place in 2014, showing microgranuloma and non-necrotizing granuloma. In 2015, moderate to severe portal and lobular granulomatous inflammation and necrosis of hepatocytes were detected. The last available liver biopsy was in 2018, and after treatment with TNF alpha inhibition showed no granulomatous lesions but lymphocytic infiltrations. Increasing splenomegaly indicated an insufficient clinical effect on PH and splenectomy was conducted (patient #5). Elevated portal pressure caused massive blood loss intraoperatively, requiring resuscitation and mass transfusion. Histology of splenic tissue revealed a nodular lymphocyte predominant Hodgkin Lymphoma in this patient.

### Immunological parameters

While mean immunoglobulin, B- and T- cell levels did not differ significantly, all CVID+PH patients had reduced levels of naïve CD45RA+CD4+ T-cells (<15%) with a mean of 6,2% and a median of 5%. EURO class classification type B- (absent or greatly reduced B-cells) was present in 5/11 (45%) of the CVID PH+ group and in only 7/185 (4%) in the CVID patients without PH (p-value < 0.00001). Increased levels of activated B cells (CD21lowCD38low) resulted in a high number of CVID+PH being categorized as EuroClass smB-21low, but neither the classification (EUROclass smB-21low: 45% vs 34%; p-value: 0.417) nor the levels of activated B-cells differed between the CVID+PH and the unaffected CVID patients (see **Supplementary Table S1**).

**Figure 1:**
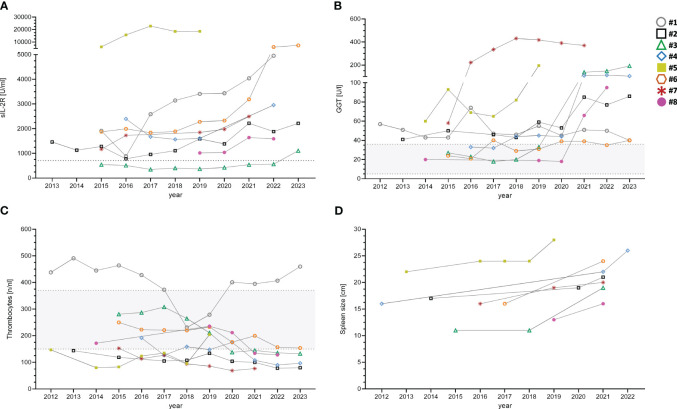
Longitudinal data of eight CVID+PH patients for sIL-2R **(A)**, GGT **(B)**, thrombocytes **(C)** and spleen size **(D)**. Gray shaded areas show standard ranges for indicated laboratory values. sIL-2R, soluble interleukin 2 receptor; GGT, gamma-glutamyl transferase.

sIL-2R was found to be significantly increased in the CVID+PH group (4105 vs U/ml vs. 1162 U/ml; p-value: 0.00026). Long-term data on the kinetics of sIL-2R was available for 8/11 patients, covering the time from CVID diagnosis until the development of PH with a median follow-up of 90 months. The mean was calculated for all parameters with > 1 available data points per analyzed year (see **Figure 1A**, **Table 5**).

**Table 5:** Immunological parameters in CVID patients with portal hypertension (CVID+PH) and CVID patients without portal hypertension (CVID).

	CVID+PH (n: 11)	CVID (n: 185)	p-value
IgG (mean in g/l) before IgRT	1.69	2.41	0.86502
IgA (mean in g/l)	0.06	0.08	0.6818
IgM (mean in g/l)	0.11	0.35	0.34722
sIL-2R (mean in U/ml)	4105	1162	0.00026
CD4+ (mean per nl)	0.62	0.62	0.42952
CD8+ (mean per nl)	0.65	1.2	0.68916
CD19+ (mean per nl)	0.18	0.2	0.242
NK cells (mean per nl)	0.15	0.14	0.56192
*B cell subsets*			
naïve B cells (mean in % of CD19+)	67.9	67	1
class switched memory B cells (mean in % of CD19+)	0.8	1.5	0.9681
activated B cells (CD21lowCD38low) (mean in % of CD19+)	21	10.4	0.5485
transitional B cells (mean in % of CD19+)	3.9	4.1	0.74896

The p-value was calculated using the Mann-Whitney-U test (IgRT= immune globulin replacement treatment). CVID, Common Variable Immunodeficiency Disorder; NK cell, Natural killer cells; sIL-2R, Soluble interleukin 2 receptor.

### Other parameters

GGT was elevated in 10/11 CVID+PH patients, while mildly elevated AST/ALT (< 2.5-fold increase of transaminases) were present in six patients. Two patients presented with slightly elevated bilirubin and INR/aPTT. PCR testing for hepatitis B, C, and E was negative in 10/10 patients and autoantibodies (ANA, AMA, and AMA-M2) were negative in 7/7 tested patients (see **Supplementary Table S1**).

Long-term follow-up demonstrated an increase of GGT over time in 7/8 patients with PH (see **Figure 1B**). The mean was calculated for all parameters with > 1 available data points per analyzed year.

Conversely, levels of thrombocytes showed reduced levels in 8/11 CVID+PH patients (see **Supplementary Table S1**). All three patients with normal thrombocytes were splenectomized in the past. A gradual decrease over time was observed in 6/8 CVID+PH patients in long-term follow-up, with the remaining two stable patients being splenectomized (see **Figure 1C**).

### Long-term follow-up of extra-hepatic non-infectious organ manifestations

Long-term follow-up data on spleen imagining demonstrated an increase in spleen size in follow-up of all seven CVID+PH patients with a mean increase of pole to pole distance of 41% (see **Figure 1D**). Longitudinal data on pulmonary function testing was available for 8 patients, showing stable diffusion capacity (KCO) in 7/8 (88%). Only patient #3 developed a progressive pulmonary insufficiency after severe pneumonia due to *Pneumocystis jirovecii* pneumonia. Imaging follow-up of lymphadenopathy was available for eight patients, indicating stable lymphadenopathy in seven (88%) (see **Table 6**).

**Table 6:** Longitudinal data on splenomegaly, lymphadenopathy, and pulmonary diffusion capacity for CO corrected for alveolar volume (DLCO/VA).

	Pulmonary diffusion capacity for CO corrected for alveolar volume (DLCO/VA)	Lymphadenopathty	Splenomegaly
**CVID+PH (#1)**	stable	Stable	increasing
**CVID+PH (#2)**	stable	Stable	increasing
**CVID+PH (#3)**	decreasing	Stable	increasing
**CVID+PH (#4)**	stable (KCO in 2016: 75% and in 2023: 65%)	Stable	increasing
**CVID+PH (#5)**	stable	Stable	increasing
**CVID+PH (#6)**	stable	Progressive	increasing
**CVID+PH (#7)**	stable	Stable	increasing
**CVID+PH (#8)**	stable	Stable	increasing

CVID, Common Variable; DLCO/VA, diffusing capacity divided by the alveolar volume; KCO, Carbon monoxide transfer coefficient; PH, Portal hypertension.

## Discussion

In the present study, we report the clinical, immunological, and histological findings of 11 patients with significant PH as hepatic involvement in a cohort of 196 CVID patients. Relevant PH was defined by the presence of at least one of the following clinical manifestations: non-malignant ascites, esophageal varices, or PH gastropathy. In our cohort, 5.6% of all adult CVID patients met these defining criteria, thereby confirming previous data on the prevalence of this hepatic complication (4, 11). The clinical burden of PH in our cohort was high. The occurrence of spontaneous bacterial peritonitis (SBP) and gram-negative sepsis shortly after TIPS implantation highlights the increased risk for infections in this patient population (17) and advocates for the early use of antibiotic prophylaxis. The mortality rate in patients with CVID+PH was higher than in unaffected CVID patients (18% vs 6%), but lower than the previously reported frequency of 28%-42% (4, 5), which may be related to the shorter observation period. Although reported as a late-onset complication, it is important to communicate that both fatal cases in our CVID+PH group occurred at a very young age (patients were deceased at 29 and 34 years).

We confirm a high association with other non-infectious manifestations in CVID+PH patients, most significantly with the occurrence of GLILD, other non-pulmonary granulomatous lesions, splenomegaly, and lymphadenopathy (4, 5, 9, 18, 19). The higher frequency of affected female patients (82%) and the shorter interval of symptom onset of PH in our cohort differ slightly from previous reports.

The most frequent histological abnormalities in CVID-associated liver disease are nodular regenerative hyperplasia-like changes (3–5, 7, 8, 13, 14). In our cohort, histopathology of the liver was available for nine patients, mostly showing an NHR-LC pattern, but also (sinusoidal) lymphocytic infiltration, mild portal or porto-septal fibrosis, and epitheloid granulomas.

In CVID patients with liver fibrosis, Crotty et al. reported a distinct pattern of pericellular fibrosis (13). In general, liver fibrosis results from collagen remodeling and the accumulation of extracellular matrix (ECM) (20). ECM can further be subdivided into the endothelial basement, mainly consisting of laminin and collagen type IV, VIII, and X, and the interstitial matrix, which is composed of the fibrillar collagens type I, II, and III. The process of tissue remodeling may also be detected in systemic circulation. Changes in the pericellular basement membrane were reported to correlate with PRO-C4 and other markers in serum of mild-to-moderate alcoholic and non-alcoholic liver disease, while PRO-C3, as a marker of interstitial matrix collagen type III, appears to correlate with progressed stages of liver fibrosis (21–23). Interestingly, PRO-C4 was recently reported to be elevated in CVID patients with non-cirrhotic PH (4).

Immune dysregulation in CVID may result from B-cell (24–26) and/or T-cell dysregulation (27, 28). In CVID, intra-sinusoidal T lymphocytes were proposed to be involved in NRH-LC pathogenesis (13). In addition to hepatic T cell receptor clonality analysis in NHR suggesting targeting of sinusoidal endothelial cells, hepatocytes were also found to show a strong overexpression of IFN gamma mRNA, proposing a pathophysiological role of cytotoxic T lymphocytes in tissue (12). Histopathological work-ups in the current study also observed T lymphocytes in the majority of examined liver tissue. However, the precise mechanisms of T-cell-mediated liver fibrosis in CVID patients remain unclear. In addition, the primary cause of T lymphocyte migration to the liver of CVID patients also remains unknown and changes in the intestinal microbiome and recurrent bacterial translocation (5, 29) or rare viral infections have been proposed (30). Larger histological cohorts are required to further examine how different histological patterns in CVID patients are related to disease stages and possible pathomechanisms.

Immunological data in peripheral blood revealed reduced levels of naïve CD45RA+CD4+ T-cells in all CVID+PH patients, thereby confirming the recent observation of lower naïve CD4+ cells in CVID patients with PH and NRH-LC (17). We also observed a higher frequency of B-lymphocytopenia (EUROclass B-) in the CVID+PH patients than in the control group of the other 185 CVID patients (45% vs 4%; p-value < 0.00001). Lower levels of B-lymphocytes in patients with CVID-associated liver diseases have been previously reported (5, 8). Although there is an association between elevated levels of activated CD21low B cells and the occurrence of non-infectious manifestations (28, 31), we did not observe any significant differences in the B-cell subsets, in particular, we did not find a higher frequency of patients with Euroclass smB-21low or elevated levels of activated B cells (CD21lowCD38low). Of note, NHR was recently reported as a frequent late-onset hepatic complication in patients with X-linked agammaglobulinemia (XLA) (32), suggesting a T-cell-driven pathomechanism in this liver disease.

Early non-invasive detection of liver disease is key, however, apart from relatively unspecific changes in liver enzymes, GGT, and platelets, there are currently no established biomarkers for hepatic manifestations in CVID patients. Transaminases may be constantly or intermittently elevated (9), and our data also confirm that normal liver enzymes cannot rule out liver manifestation in CVID. Globig et al. proposed PRO-C4, a serum biomarker of liver fibrosis and collagen formation in CVID patients (4) and Lima et al. reported higher levels of beta-2-microglobulin in CVID patients with liver-spleen-axis abnormalities in comparison to those patients without hepato-splenic changes (5). In the present study, we confirm the association of low platelets and elevated GGT with PH in CVID patients.

Due to the multi-organ involvement with many non-infectious manifestations occurring simultaneously, common underlying pathomechanisms have been proposed in CVID patients (28, 31, 33). However, the frequent co-occurrence of different organ complications is hampering the identification of specific risk factors and biomarkers that could be related exclusively to the involvement of a single non-infectious manifestation. By analyzing longitudinal data in our patient cohort, we show for the first time the dynamics of different biomarkers (GGT, platelets, sIL-2R) and imaging (spleen size) in CVID patients both before the development and under the clinical manifestation of PH.

While the small sample size limits the interpretation, looking at longitudinal data might nevertheless allow us to get a slightly more granular glimpse of the development of portal hypertension in CVID. Eight longitudinally followed CVID+PH patients presented at the time of CVID diagnosis with a multi-organ combination of splenomegaly, lymphadenopathy, and GLILD. Over time, lymphadenopathy and GLILD remained stable in 7/8 patients, while imaging revealed a gradually increasing splenomegaly, and analysis of blood markers showed an elevation of sIL-2R in addition to a decrease in platelets and an increase of GGT.

However, elevated sIL-2R levels cannot be regarded as a specific marker for hepatic involvement. In addition to significantly higher levels of soluble IL-2 receptor (sIL-2R) in the CVID+PH patients of our cohort, we could also confirm elevated levels in patients with GLILD (manuscript in preparation) (34). Therefore, sIL-2R should be considered a general marker of T cell activation (35–37). Our longitudinal data from CVID+PH patients expand that spectrum and suggest that it may, in combination with other markers, also hint at progressive PH in CVID patients.

In addition to biomarkers for screening of hepatic manifestations, imaging plays a pivotal role. Our observation of mostly normal findings in conventional sonography, including normal velocity of portal venous flow in (82%), are in line with the so far largest single center analysis (4), however, other studies reported sonographic abnormalities in approximately 50%-66% of CVID patients with NRH-LC (12, 14) and detected portal vein enlargement in 25% of patients with primary antibody deficiency (11). Of note, significantly elevated HVPG (> 10 mmHg) was detected in 6/6 patients despite normal velocity in portal trunk, suggesting an insufficient sensitivity of this non-invasive examination for the detection of non-cirrhotic PH in CVID patients.

While our longitudinal data also indicate that increasing spleen size should raise high suspicion of the presence of PH, other causes should always be considered. This is exemplified by patient #5, where histology of splenic tissue after splenectomy due to massive splenomegaly with organ compression revealed in addition a nodular lymphocyte predominant Hodgkin Lymphoma.

Inspired by the applicability of transient elastography in other chronic liver diseases (38–40), attention has also been drawn to its diagnostic use for identifying hepatic manifestations in CVID patients (4, 18, 19). All the CVID+PH patients in this study with proven PH (HVPG ≥ 10 mmHg) expressed increased rates of liver stiffness, thereby confirming previous reports and suggesting the early use of this methodology in CVID cases with suspected liver involvement (4, 18). However, transient elastography is not widely available and harbors technical challenges, such as in obese patients. Additionally, liver stiffness is not exclusively influenced by liver inflammation, but also by prandial status, biliary obstruction, or cardiac output (41).

Our study has important limitations. With an estimated prevalence of 5%-6% of CVID patients suffering from PH, our sample size is underpowered and the median follow-up time is not sufficient for a solid evaluation of long-term morbidity and mortality of PH in CVID. In order to better understand the pathomechanisms of liver disease in CVID, future studies would certainly benefit from a standardized in-depth analysis of tissue samples, including detailed immunohistochemical staining of lymphocytes, and should also include a more profound assessment of pericellular fibrosis.

Currently, no specific EMA- or FDA-approved treatment for CVID patients with PH due to NRH-LC is available. Data on corticosteroids are variable (12, 42) and there are only a few reports on the use of anti-TNFa treatment (43, 44). In our cohort, patient #5 showed a good response to anti-TNFa treatment on granuloma formation, however, overall liver disease progressed. Outcome data after liver transplantation are also variable, with the re-occurrence of NRH-LC in the transplanted organ in CVID patients (45–48) as well as in patients without inborn errors of immunity (49). Despite the elevated morbidity of CVID patients with non-cirrhotic PH, liver transplantation remains a challenging option. First, this is because the clinical severity of liver disease is mainly assessed by the MELD score and most CVID patients with non-cirrhotic PH score too low for listing (50). Second, many centers are reluctant to accept CVID patients due to preexisting comorbidities. Two of our patients died following acute infection. The role of HSCT remains unclear but it was reported that the presence of NRH-LC may affect outcomes after stem cell transplantation (51).

Our single center analysis of PH in CVID patients could confirm the high degree of clinical burden and an estimated prevalence of 5%-6%. Our data support a role for transient elastography during early screening and reveal that non-invasive ultrasound-based assessment can be normal despite significantly elevated HVPG. In addition, our longitudinal data suggest that the combination of increasing spleen size, increasing sIL2-R and GGT values, and gradually reducing platelets should raise high suspicion of the presence of PH. Currently, consensus recommendations for diagnostics and management of liver disease in CVID patients are rare (14) and larger observational and interventional studies as well as international clinical guidelines are urgently needed.

## Data availability statement

The original contributions presented in the study are included in the article/**Supplementary Material**. Further inquiries can be directed to the corresponding author.

## Ethics statement

The studies involving humans were approved by ethics commitee of Charité (EA4/112/19), Berlin, Germany. The studies were conducted in accordance with the local legislation and institutional requirements. The participants provided their written informed consent to participate in this study. Written informed consent was obtained from the individual(s) for the publication of any potentially identifiable images or data included in this article.

## Author contributions

LH: Conceptualization, Data curation, Formal analysis, Investigation, Methodology, Supervision, Writing – original draft, Writing – review & editing. SS: Visualization, Writing – review & editing. KW: Writing – review & editing. MS: Writing – review & editing. CK: Writing – review & editing. CS: Writing – review & editing. AF: Conceptualization, Project administration, Writing – review & editing.

## Glossary

**Table:**

ALT	alanine transaminase
AMA	anti-mitochondrial antibody
ANA	antinuclear antibody
aPTT	activated partial thromboplastin time
AST	aspartate aminotransferase
CTLA4	cytotoxic T-lymphocyte-associated protein 4
CVID	common variable immunodeficiency disorder
CVID+PH	CVID with portal hypertension
ECM	extracellular matrix
ESID	European Society for Immunodeficiencies
GGT	gamma glutamyl transferase
HSCT	hematopoietic stem cell transplantation
HVPG	hepatic venous pressure gradient
IgA	immunoglobulin A
IgG	immunoglobulin G
IgM	immunoglobulin M
INR	international normalized ratio
ITP	immune thrombocytopenia
KCO	carbon monoxide transfer coefficient
kPa	kilo Pascal
NFKB1	nuclear factor kappa B subunit 1
NGS	next-generation sequencing
NRH	nodular regenerative hyperplasia
NRH-LC	nodular regenerative hyperplasia-like changes
PCR	polymerase chain reaction
PH	portal hypertension
PID	primary immunodeficiency disease
sIL- 2R	soluble interleukin 2 receptor (sCD25)
SBP	spontaneous bacterial peritonitis
TIPS	transjugular intrahepatic portosystemic shunt
TNFa	tumor necrosis factor alpha
WES	whole exome sequencing
XLA	X-linked agammaglobulinemia
